# Occupational risk of COVID-19 across pandemic waves: a two-year national follow-up study of hospital admissions

**DOI:** 10.5271/sjweh.4056

**Published:** 2022-10-29

**Authors:** Jens Peter Ellekilde Bonde, Lea Sell, Johan Høy Jensen, Luise Mølenberg Begtrup, Esben Meulengracht Flachs, Kristina Jakobsson, Christel Nielsen, Kerstin Nilsson, Lars Rylander, Kajsa Ugelvig Petersen, Sandra Søgaard Tøttenborg

**Affiliations:** 1 Department of Occupational and Environmental Medicine, Bispebjerg and Frederiksberg Hospital, Copenhagen University Hospital, Copenhagen, Denmark.; 2 Department of Public Health, University of Copenhagen, Copenhagen, Denmark.; 3 School of Public Health and Community Medicine, Sahlgrenska Academy, University of Gothenburg, Sweden.; 4 Occupational and Environmental Medicine, Sahlgrenska University Hospital, Gothenburg, Sweden.; 5 Division of Occupational and Environmental Medicine, Department of Laboratory Medicine, Lund University, Lund, Sweden.; 6 Division of Public Health, Kristianstad University, Kristianstad, Sweden.; 7 Clinical Pharmacology, Pharmacy and Environmental Medicine, Department of Public Health, University of Southern Denmark, Odense, Denmark.

**Keywords:** cohort study, epidemiology, industry, ISCO-08, job, NACE, SARS-CoV-2

## Abstract

**Objective:**

Assuming that preventive measures to mitigate viral transmission of SARS-CoV-2 at the workplace may have been improved in the course of the COVID-19 pandemic, we examined the occupational risk of COVID-19 related hospital admission across the four pandemic waves in Denmark between week 8, 2020, and week 50, 2021.

**Methods:**

The study included 4416 cases of COVID-19 related hospital admissions among 2.4 million Danish employees aged 20–69 with follow-up in 2020 through 2021. At-risk industrial sectors and a reference population were defined a priory by a job-exposure matrix on occupational risk for COVID-19. Incidence rate ratios (IRR) and potential effect modification by pandemic wave were computed with Poisson regression adjusted for demographic, social and health factors including completed COVID-19 vaccination.

**Results:**

We observed an overall elevated relative risk in four of six at-risk industrial sectors, but the pandemic wave only modified the risk among healthcare employees, where the excess risk from a high initial level declined to background levels during the latest waves in models not adjusting for COVID-19 vaccination. In social care, education and transport, the elevated risk was not modified by pandemic wave.

**Conclusion:**

Danish healthcare employees were to some extent protected against occupational transmission of SARS-CoV-2 during the two last pandemic waves even though the absolute risk conferred by occupation may not have been eliminated. Early vaccination of this group seems not to be the only explanation. The risk in other sectors remained elevated indicating a need to revisit preventive measures.

The workplace has contributed strongly to the spread of SARS-CoV-2 (severe acute respiratory syndrome corona virus 2) during the COVID-19 pandemic. Clusters originating in the occupational setting have been extensively reported (1), and several follow-up studies have demonstrated substantially increased risk for infection (2), COVID-19-related hospital admission (3) and death (4) in numerous occupations. Until vaccines became available, safety measures to prevent viral transmission at the workplace have – in addition to (forced) closure and home working – mostly included simple generic recommendations such as social distancing and masks issued by WHO and national health authorities. So far, few studies have addressed the effectiveness of these measures regarding the occupational setting (5–7).

We hypothesized that the occupational risk of COVID-19 reached background levels as regulations, recommendations, and training in use of the most appropriate personal equipment might have become implemented still more effectively during the pandemic. We addressed this hypothesis by examining the occupational risk of COVID-19 related hospital admission in several industrial sectors across the pandemic waves in Denmark in 2020 through 2021.

## Methods

### Population and data

We used a nationwide cohort of all Danish employees aged 20–69 years with registry data on job and industry codes in December 2019. This cohort is a subset of the DOC*X cohort (Danish Occupational Cohort with eXposure data) (8). Occupations were classified according to the Danish version of the International Standard Classification of Occupations [DISCO-08 (32)] and industries according to the Statistical Classification of Economic Activities in the European Communities [DB07 (33)].

At-risk occupations and a reference group were defined by an expert rated job-exposure matrix (JEM) with eight domains addressing risk of SARS-CoV-2 transmission at the workplace, preventive measures and job insecurity, each rated from 0 (low exposure) to 3 (high exposure). This JEM was developed independently of this study (9). Occupations with a JEM sum score >12 (on a scale from 0–24) within each of six industrial sectors with an average JEM sumscore >12 at the 2-digit DB07 level were *a priory* considered exposed. The JEM expert group considered an occupation at no risk (JEM sumscore 0) if employees were working from home or not working, if the proportion of employees with income insecurity because of the pandemic was <1% and if migrant workers constituted <1%. Thus, the 50 4-digit DISCO-08 occupations with a JEM sumscore of 0 constituted the reference group (supplementary material, www.sjweh.fi/article/4056, table S1).

The outcome was defined as hospital admission of ≥12 hours in combination with a positive SARS-CoV-2 polymerase chain reaction (PCR) test up to 14 days prior to admission. Outcome data and individual-level demographic, social and health information data, including date of COVID-19 vaccinations (Pfizer BioNTech, Moderna, Janssen or Astra Zeneca), were retrieved from nationwide public registries hosted by Statistics Denmark and the Danish Health Data Authority.

While data on industrial sector at the 2-digit DB07 level were available for all employees, the DISCO-08 codes at the 4-digit level were missing for 13.8% of the population and data on education for 2.0%. Otherwise, data were complete.

Details on the cohort and its key variables are provided in Bonde et al (10).

### Interventions targeting workplaces in Denmark

**Figure 1:**
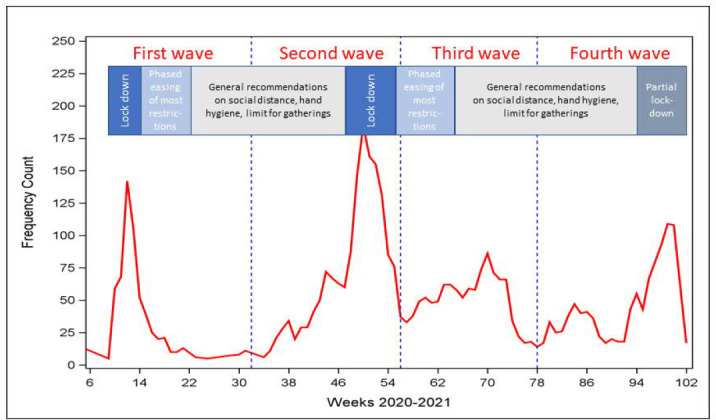
Covid-19 related hospital admissions among employees age 20-69 in Denmark in 2020 and 2021. **Lockdown:** Non-essential public employees are working from home or sent home from work with salary (essential public workers include health and social care, transportation, police, prisons, emergency functions). Children daycare institutions, primary schools, high schools, boarding schools, higher education and universities (ie, educational sector), courts, churches, libraries, museums, theaters, cinemas, zoological gardens, and other cultural institutions are closed. Private sector employees encouraged to work home if possible. International travel, hotels, restaurants, night clubs, fitness centers and sport closed. Shopping centers and retail sales except groceries and drug stores closed. Liberal services as hairdressers and beauty shops closed. **Partial lockdown:** Temporary and less restrictive lockdowns of the childcare, education, entertainment and public non-essential institutions. Gradual easing of restrictions: discontinuation of restrictions in sequential phases – opening of daycare and primary schools grade 0-5 first and night clubs, sport events and cultural events with large gatherings last. Other measures: face mask mandatory in public transportation from 22.8.2020 and in shops and education from 29.10.2020.

On 28 January 2020, the Danish Health Authority issued generic recommendations to minimize the risk of infection by social distancing and hand hygiene. The first Danish citizen tested positive for SARS-CoV-2 on 7 February 2020. Use of face masks in public transport and indoor locations became mandatory on 22 August 2020. COVID-19 vaccinations started on 27 December 2020 and were preferentially offered to healthcare workers, elders, and vulnerable persons (supplementary table S2). Periods of lockdown and gradual societal reopening are provided in figure 1. Outdoor use of face masks and curfews were not enforced at any time during the pandemic in Denmark.

### Statistical analysis

The study used a follow-up period from week 8 in 2020 through week 50 in 2021 divided into four pandemic waves delineated by midpoints of the troughs between peaks of COVID-19 related hospital admissions in Denmark (figure 1). Incidence rate ratios (IRR) with 95% confidence intervals (CI) for COVID-19 related hospital admission were computed by Poisson regression. Hospital admissions in each at-risk industrial sector were compared with the occurrence in the reference group across all epidemic waves and in each of the four waves (between industrial sectors comparisons). Moreover, to examine development of risk within industrial sectors, we computed the risk in waves 2, 3 and 4 referenced with wave 1 (within industrial sector comparisons). The time unit was a week, and follow-up was censored at the first of COVID-19-related hospital admission, death, emigration, retirement, or the end of week 50 in 2021. Missing values for DISCO-08 codes and education were kept as separate categories in all analyses.

Between-group comparisons were adjusted by a fixed set of baseline variables according to the disjunctive confounder variable selection criteria (11): sex, age, duration of education, country of origin, geographical area and chronic diseases (details are given in footnote to table 1). These variables were strongly associated with COVID-19 hospital admission in earlier analyses (11). Within-group comparisons across pandemic waves were not adjusted since most of the mentioned covariates are fixed across short time spans.

**Table 1:** Incidence rate ratio (IRR) with 95% confidence intervals (CI) for COVID-19 related hospital admission within at-risk industrial sectors^1^across pandemic waves and between at-risk industrial sectors in comparison with a COVID-19 job exposure matrix (JEM)-based reference group.

Occupations Within and between group difference	DB07 code	All waves			Wave 1 week 8–32 2020 (Alpha variant)			Wave 2 week 33–52 2021, 1-4 2021 (Alpha variant)			Wave 3 week 5–26 2021 (Beta variant)			Wave 4 week 27–50 2021 (Delta variant)			Test for trend ^2^/interaction ^3^
					
		N	IRR	95% CI	N	IRR	95% CI	N	IRR	95% CI	N	IRR	95% CI	N	IRR	95% CI	P-value
Healthcare	85	314			102			120			42			50			
Within ^2^			-	-		1.00			1.23	1.0-1.6		0.47	0.3-0.7		0.52	0.4-0.7	<0.0001
Between ^3^			1.66	1.4-1.9		3.59	2.7-4.9		1.64	1.3-2.1		0.94	0.7-1.3		1.19	0.9-1.7	<0.0001
Social care	87–88	628			95			251			117			165			
Within ^2^			-	-		1.00			2.76	2.2-3.5		1.41	1.1-1.9		1.82	1.4-2.4	<0.0001
Between^3^			1.40	1.2-1.6		1.51	1.1-2.1		1.45	1.2-1.8		1.09	0.9-1.4		1.58	1.2-2.0	0.069
Education	86	226			22			89			58			57			
Within ^2^			-	-		1.00			4.24	2.7-6.8		3.02	1.9-4.9		2.72	1.7-4.5	<0.0001
Between ^3^			1.31	1.1-1.5		0.80	0.5-1.3		1.30	1.0-1.7		1.43	1.1-2.0		1.60	1.2-2.2	0.185
Transport	49-51	128			17			42			46			23			
Within ^2^			-	-		1.00			2.60	1.5-4.6		3.10	1.8-5.5		1.44	0.8-2.7	<0.0001
Between ^3^			1.85	1.5-2.3		1.69	1.0-2.9		1.44	1.0-2.0		2.73	1.9-3.9		1.77	1.1-2.8	0.051
Retail sales	46	120			12			44			24			40			
Within ^2^						1.00			3.83	2.0-7.3		2.29	1.1-4.6		3.49	1.8-6.7	<0.0001
Between ^3^			1.01	0.8-1.2		0.93	0.5-1.7		1.09	0.8-1.5		0.84	0.5-1.3		1.13	0.8-1.6	0.037
Service trades	55, 56, 81, 93, 96	213			21			83			63			46			
Within ^2^			-	-		1.00			4.16	2.6-6.7		3.46	2.1-5.7		2.32	1.4-3.9	<0.0001
Between ^3^			0.93	0.8-1.1		0.78	0.5-1.3		0.97	0.8-1.2		1.09	0.8-1.5		0.78	0.6-1.1	0.160
Others^4^		1535			216			531			422			366			
Within ^2^			-	-		1.00			2.58	2.2-3.0		2.24	1.9-2.6		1.78	1.5-2.1	<0.0001
Between ^3^			0.94	0.9-1.0		0.91	0.7-1.2		0.80	0.7-0.9		1.09	0.9-1.3		1.08	0.9-1.3	0.065
Missing DISCO-08 code		693			76			224			183			210			
Within ^2^			-			1.00			3.10	2.4-4.0		2.77	2.1-3.6		2.92	2.3-3.8	<0.0001
Between ^3^			1.15	1.0-1.3		0.94	0.7-1.3		0.97	0.8-1.2		1.25	1.0-1.6		1.48	1.2-1.9	<0.0001
JEM-based reference group ^3^		559	1.0		83	1.00		224	2.84	2.2-3.7	133	1.84	1.4-2.4	118	1.49	1.1-2.0	<0.0001

1 Industrial sectors at the 2-digit DB07 level with a higher likelihood of occupational SARS-CoV-2 exposure according to an expert rated COVID-19 job exposure matrix (sumscore >12, range 0-24) (9).

2 Crude risk across pandemic waves within each group of occupations relative to the occurrence in the first wave. P-values for test of non-linear trend in risk across waves (within industrial sector comparisons).

3 Wave-specific risk adjusted for sex, age (10 year groups), duration of education at baseline (5 groups), number of hospital admissions for one or more of 11 chronic diseases in the 10 years preceding start of the pandemic (0, 1, >1), country of origin (4 categories) and geographical region (5 groups) in an at-risk industrial sector compared with a COVID-19 JEM reference group (9) (all employees with low likelihood of occupational SARS-CoV-2 exposure (sumscore for all eight rated measures = 0)). P-values for interaction testing if the pandemic wave modified the effect of industrial sector on risk of COVID-19 hospital admission (between industrial sector comparisons).

4 Employees in other industrial sectors and employees with COVID-19 JEM sumscore ≤12.

To test if the pandemic wave modified the occupational risk of COVID-19 related hospital admission, in addition to the main effects, we included an interaction-term (industrial sector×pandemic wave) in the between- group regression models for each of the six industrial sectors.

To account for non-monotonic trends across the four pandemic waves within each of the at-risk industrial sectors, the wave variables taking the value of integers 1–4 were introduced as numeric linear and squared terms in the regression models of within-group change over time. These analyses were performed because the data indicated an increased risk followed by a decline in all industrial sectors

Completed COVID-19 vaccination may be a mediator of the effect of exposure (at-risk occupation) on COVID-19 occurrence as well as a confounder since vaccination was not offered at random. Therefore, in supplementary analyses of between-group comparisons in waves 3 and 4, we included completed vaccination as a time-varying variable.

All analyses were carried out in SAS 9.4 (SAS Institute, Cary, NC, USA) on a platform at Statistics Denmark.

## Results

The average incidence of COVID-19 related hospital admission among Danish employees aged 20–69 years across the first two years of the pandemic was 19.2 admissions per million person-weeks with peaks during spring and winter 2020, and spring and autumn 2021 (figure 1). In the age range 20–40 years, the incidence was highest among women and increased during the pandemic. In the age range 41–69 years, the incidence was highest among men and decreased in both sexes during the pandemic (supplementary table S3).

*Within-group analyses*. In all sectors including the reference group, the risk of COVID-19 related hospital admission increased substantially from the first wave in spring 2020 to the second wave and then declined during the subsequent waves (table 1). The trend test allowing for non-monotonic change across waves were significant for all sectors. Despite the declining trend from the second wave onwards, the risk remained elevated above the initial level except in healthcare workers, where the risk was almost halved during the last two waves.

*Between-group analyses and interaction*. The overall average risk was elevated in all sectors in comparison with the reference population except retail sales and various services (table 1), but the risk relative to the reference population was only statistically significantly modified by the pandemic wave among healthcare and retail sales employees. Among healthcare workers, the initial high risk declined to the reference level during the two last waves. Among retail sales employees, risk was not increased in comparison with the referents in any wave, but within retail sales employees, it was strongly increased in the last three waves indicating that in this group, the risk was exceptionally low in the first wave.

Healthcare workers were offered vaccination free of charge earlier than other occupational groups. While 25% of healthcare workers had completed vaccination by the fifth week of 2021, it took almost half a year before 25% of the referent population had completed vaccination (supplementary table S2). Inclusion of COVID-19 vaccination status in the between-group regression models for pandemic waves 3 and 4 resulted in a substantially increased IRR among healthcare employees compared to models not including vaccination (supplementary table S4). For other sectors changes were minor.

## Discussion

In this follow-up study of COVID-19-related hospital admissions among Danish employees, we observed an overall elevated average risk in four of six a priori JEM-defined at-risk industrial sectors, but the pandemic wave only modified the risk in healthcare employees, where the risk from a high initial level declined to background levels during the latest waves. In social care, the educational and transport sector, the elevated risk was not modified by pandemic wave.

*Limitations*. To obtain robust risk estimates and enough COVID-19 admissions to allow interaction analysis, we examined risk in large industrial sectors. However, specific occupations within these sectors may have risk profiles that deviate from the overall average. Moreover, we were unable to account for people changing their occupation during follow-up, but since the follow-up period was short, this problem is likely to be minor. Some COVID-19-related hospital admissions have been due to other disorders, but an analysis of discharge diagnoses for a subset of the population indicates that during the observed period, this proportion was only about 2–3% (11). Analyses were adjusted for a fixed set of baseline characteristics that are strong predictors of COVID-19 related hospital admission (11), but we were unable to account for all potentially confounding factors – for instance risk related to commuting by public transportation.

*Context and implications*. Only a few studies have examined the development of occupational risk across pandemic waves (5, 7, 12) and no earlier studies have presented data directly comparable to those presented here. In any case, the development of the pandemic and associated occupational risks must be understood in the specific context of a given country. In Denmark, the apparent elimination of the relative occupational risk in healthcare during the second year of the pandemic is only partly explained by COVID-19 vaccination – that was provided early for this group of employees – because the relative risk seemed to be reduced before the majority of healthcare workers had completed vaccination. Improved access to appropriate personal protective equipment (13) and adherence with infection control guidelines may also have contributed to reducing the risk of virus acquisition at the workplace among healthcare providers (5, 14–16) who are professionally trained in aseptic procedures (17). In any case, it should be acknowledged that the effect of vaccination on the relative risk of COVID-19 among healthcare workers may be temporary as even low-level exposed occupational groups become vaccinated too. Therefore, compliance with preventive guidelines should not be relaxed. Unfortunately, the data do not indicate that safety measures improved during the pandemic in other at-risk industrial sectors. On the contrary, the relative risk in the education and transportation sectors was higher in the later pandemic waves compared to the first, which may reflect relaxing the strict close-down of society in the first wave in which schools were closed for all ages and transportation was kept to a minimum. Spread of virus mutants with greater transmissibility such as SARS-CoV-2 B.1.1.7 [the British Beta-variant (18)], which became the most prevalent in Denmark in February 2021 (19), may have decreased the effectiveness of safety measures.

### Concluding remarks

Danish healthcare workers were at least partially protected against COVID-19-related hospital admission during the two last pandemic waves. Nevertheless, strict adherence to infection control measures at the workplace is still needed. The elevated risk in social care, education and transport remained at an elevated level throughout the pandemic and indicates a need to reinforce the use of preventive measures and maintain vaccination campaigns in these sectors.

## Supplementary material

Supplementary material
