# Supplementary material for: Occupational risk of COVID-19 across pandemic waves: a two-year national follow-up study of hospital admissions
Source: Scand J Work Environ Health. 2022 Oct 29;48(8):672–7. doi: 10.5271/sjweh.4056 (PMC10546612; doi:10.5271/sjweh.4056)
Supplement: Supplementary material [file SJWEH-48-672-S001.pdf]

**Occupational risk of COVID-19 across pandemic waves: a two-year national follow-up study of hospital admissions**[1]

by Jens Peter Ellekilde Bonde, MD,[2] Lea Sell, PhD, Johan Høy Jensen, PhD, Luise Mølenberg Begtrup, PhD, Esben Meulengracht Flachs, PhD, Kristina Jakobsson, PhD, Christel Nielsen, PhD, Kerstin Nilsson, PhD, Lars Rylander, PhD, Kajsa Ugelvig Petersen, PhD, Sandra Søgaard Tøttenborg, PhD

1. Supplementary material
2. Correspondence to: Jens Peter Bonde, Department of Occupational and Environmental Medicine, Bispebjerg and Frederiksberg Hospital, Bispebjerg Bakke 23, DK-Copenhagen 2400 NV, Denmark. [E-mail: Jens.Peter.Ellekilde.Bonde@regionh.dk]

**Supplemental table S1. Occupational groups at the 4-digit DISCO-08 level in industrial sectors with an average Covid-19 JEM score above 12[1] All occupational groups are included in analyses, but only specific occupations with > 2000 employees are listed.**

| Occupation (descending number of employees) | DISCO-08 code | N employees | JEM SUM-SCORE[1] (range 0-24) |
|---|---|---|---|
| HEALTHCARE (DB07 code 86) | - | 133 093 | 15.6 (average) |
| Nursing professionals | 2221 | 51 156 | 16 |
| Generalist medical practitioners | 2211 | 18 518 | 16 |
| Healthcare Assistants | 5321 | 12 620 | 18 |
| Dental assistants and therapits | 3251 | 8068 | 13 |
| Physiotherapists | 2264 | 6596 | 14 |
| Medical and Pathology Laboratory Technicians | 3212 | 6229 | 16 |
| Psychologists | 2634 | 5052 | 13 |
| Health Professionals Not Elsewhere Classified | 2269 | 4495 | 16 |
| Cleaners and Helpers in Offices, Hotels and Other Establishments | 9112 | 4045 | 19 |
| Dentists | 2261 | 3351 | 14 |
| Medical Imaging and Equipment Operators | 3211 | 2438 | 14 |

| | | | |
|---|---|---|---|
| Midwifery Professionals | 2222 | 2042 | 15 |
| SOCIAL CARE (DB07 code 87-88) | | 282 214 | 15.8 (average) |
| Home-based Personal Care Workers | 5322 | 81 233 | 18 |
| Early childhood educators | 2343 | 55 842 | 14 |
| Childcare workers | 5311 | 42 012 | 14 |
| Special Teaching Professionals | 2357 | 31 529 | 15 |
| Healthcare Assistants | 5321 | 27 224 | 18 |
| Nursing professionals | 2221 | 11 103 | 16 |
| Social Work and Counselling Professionals | 2635 | 4314 | 13 |
| Cleaners and Helpers in Offices, Hotels and Other Establishments | 9112 | 4178 | 19 |
| Social Work Associate Professionals | 3412 | 4175 | 13 |
| Health Professionals Not Elsewhere Classified | 2269 | 3640 | 16 |
| Primary school teachers | 2341 | 3316 | 14 |
| Physiotherapists | 2264 | 2353 | 14 |
| Kitchen Helpers | 9412 | 2184 | 17 |
| Occupation (descending number employees) | DISCO-08 code | N employees | JEM SUM-SCORE[1] (range 0-24) |
| EDUCATION (DB07 85) | | 128 780 | 14.4 (average) |
| Primary school teachers | 2341 | 72 524 | 14 |

| | | | |
|---|---|---|---|
| Secondary Education Teachers | 2330 | 15 006 | 14 |
| Early childhood educators | 2343 | 12 487 | 14 |
| Childcare workers | 5311 | 5379 | 14 |
| Cleaners and Helpers in Offices, Hotels and Other Establishments | 9112 | 3704 | 19 |
| Special Needs Teachers | 2352 | 3349 | 15 |
| Building caretakers | 5153 | 3313 | 14 |
| TRANSPORTATION (DB07 code 49-51) | | 21 120 | 18.5 (average) |
| Bus and Tram Drivers | 8331 | 10 775 | 19 |
| Car, Taxi and Van Drivers | 8322 | 2858 | 21 |
| Travel Attendants and Travel Stuarts | 5111 | 2090 | 19 |
| RETAIL AND SALES (DB07 code 45) | - | 84 832 | 18.4 (average) |
| Shop Sales Assistants | 5223 | 55 060 | 19 |
| Cashiers and Ticket Clerks | 5230 | 12 804 | 18 |
| Service Station Attendants | 5245 | 3592 | 19 |
| Shelf Fillers | 9334 | 3248 | 17 |
| ACCOMODATION, FOOD, BUILDING PERSONAL AND PROTECTIVE SERVICES, RECREATION (DB07 codes 55 ,56, 81, 93, 96) | | 100 540 | 17.2 (average) |
| Cleaners and Helpers in Offices, Hotels and Other Establishments | 9112 | 27 849 | 19 |
| Cooks | 5120 | 10 496 | 13 |
| Waiters | 5131 | 10 332 | 21 |

| | | | |
|---|---|---|---|
| Kitchen Helpers | 9412 | 7658 | 17 |
| Civil Engineering Labourers | 9312 | 6003 | 13 |
| Building caretakers | 5153 | 5660 | 14 |
| Hairdressers | 5141 | 5398 | 17 |
| Fast Food Preparers | 9411 | 3610 | 19 |
| Hotel Receptionists | 4224 | 2679 | 16 |
| Occupations, Covid-19 JEM sumscore > 0, ≤12 | - | 993 939 | 10.6 (average) |
| Missing DISCO-08 code, | - | 337 306 | - |
| JEM based reference group [1] | - | 369 341 | 0 |

[1] Likelihood of occupational SARS-CoV-2 exposure according to a population-based international expert-rated job exposure matrix that assesses four measures of number of close indoor contacts at work, two mitigation measures and two job insecurity measures, each rated on a scale from low (0) to high (3)

**Supplemental table S2. Week in 2021 with completed second Covid-19 vaccination by industrial sector considered at higher risk according to a Covid-19 job exposure matrix.**

| High-risk industrial sector | 25 percentiles | Median | 75 percentiles | 90 percentiles |
|---|---|---|---|---|
| Healthcare | 5 | 19 | 24 | 32 |
| Social care | 14 | 24 | 30 | 33 |
| Education | 25 | 28 | 31 | 33 |
| Transportation | 24 | 26 | 29 | 33 |
| Retail sales | 25 | 30 | 32 | 34 |
| Service occupations | 23 | 29 | 32 | 34 |
| Reference population | 25 | 28 | 21 | 33 |

Note: The adjusted incidence risk rate ratio for Covid-19 related admission to hospital in 2021 was 0.26 (0.20-0.34) among vaccinated compared to non-vaccinated healthcare workers.

**Supplemental table S3. Covid-19 related hospital admission incidence (number/year/million employees) by sex and age stratified by pandemic wave in Denmark 2020-21**

| Pandemic waves 2020-21 | Men, age years | | | Women, age years | | | All |
|---|---|---|---|---|---|---|---|
| Weeks | 20-40 | 41-55 | 56-69 | 20-40 | 41-55 | 56-69 | |
| 1 8 - 32 | 7.3 | 23.4 | 47.6 | 11.8 | 22.7 | 31.3 | 21.6 |
| 2 33- 56 | 25.4 | 69.9 | 133.6 | 43.1 | 48.4 | 75.9 | 60.1 |
| 3 57- 78 | 35.0 | 65.7 | 80.9 | 34.3 | 44.1 | 43.1 | 48.7 |
| 4 79-102 | 38.6 | 42.7 | 46.7 | 56.4 | 26.1 | 29.6 | 40.4 |

**Supplemental table S4. Incidence rate ratio (IRR) with 95% confidence intervals (CI) for Covid-19 related hospital admission for at-risk industrial sectors[1] with and without adjustment for Covid-19 vaccination.**

| Occupations | DB07 code | 3.wave week 5-26 2021 | | | 4. wave Week 27-50 2021 | | |
|---|---|---|---|---|---|---|---|
| | | N Covid-19 | IRR | 95% CI | N Covid-19 | IRR | 95% CI |
| Healthcare | 85 | | | | | | |
| Without adjustment[2] | | 42 | 0.86 | 0.6-1.3 | 50 | 1.26 | 0.9-1.8 |
| With adjustment[3] | | | 1.39 | 1.0-2.0 | | 1.48 | 1.0-2.1 |
| Social care | 87-88 | | | | | | |
| Without adjustment[2] | | 117 | 1.02 | 0.8-1.3 | 165 | 1.70 | 1.3-2.2 |
| With adjustment[3] | | | 1.20 | 0.9-1.6 | | 1.57 | 1.2-2.0 |
| Education | 86 | | | | | | |
| Without adjustment[2] | | 58 | 1.29 | 0.9-1.8 | 57 | 1.60 | 1.1-2.3 |
| With adjustment[3] | | | 1.30 | 0.9-1.8 | | 1.46 | 1.0-2.1 |
| Transport | 49-51 | | | | | | |
| Without adjustment[2] | | 46 | 2.73 | 1.8-4.2 | 23 | 1.98 | 1.2-3.4 |
| With adjustment[3] | | | 2.71 | 1.8-4.1 | | 1.64 | 1.0-2.8 |
| Retail sales | 46 | | | | | | |
| Without adjustment[2] | | 24 | 0.73 | 0.5-1.2 | 40 | 1.20 | 0.8-1.8 |
| With adjustment[3] | | | 0.73 | 0.5-1.2 | | 1.11 | 0.7-1.7 |
| Service trades | 55,56, 81,93,96 | | | | | | |
| Without adjustment[2] | | 82 | 1.04 | 0.7-1.5 | 63 | 0.88 | 0.6-1.3 |
| With adjustment[3] | | | 1.04 | 0.7-1.5 | | 0.76 | 0.5-1.1 |
| JEM based reference group[2] | - | 133 | 1.00 | - | 118 | 1.00 | - |

[1] Industrial groups at the 2-digit DB07 level with a higher likelihood of occupational SARS-CoV-2 exposure according to an expert rated Covid-19 job exposure matrix (sumscore >12, range 0-24) (9).
[2] Wave-specific risk adjusted for sex, age (10 year groups), duration of education at baseline (5 groups), number of hospital admissions for one or more of 11 chronic diseases in the 10 years preceding start of the pandemic (0, 1, >1), country of origin (4 categories) and geographical region (5 groups) in an high-risk industrial sector compared with a Covid-19 JEM reference group (9) (all employees with low likelihood of occupational SARS-CoV-2 exposure (sumscore for all eight rated measures = 0))
3 Adjustment for completed Covid-19 vaccination (time varying variable) offered from week 1 in 2021 in addition to the above covariates.
